# Knowledge, attitude and behaviour regarding dietary salt intake among medical students in Angola

**DOI:** 10.5830/CVJA-2015-018

**Published:** 2015

**Authors:** Pedro Magalhães, Edgar JR Sanhangala, Isildro M Dombele, Henrique SN Ulundo, Daniel P Capingana, Amílcar BT Silva

**Affiliations:** Department of Physiology, Faculty of Medicine, University Agostinho Neto, Luanda, Angola; Department of Physiology, Faculty of Medicine, University Agostinho Neto, Luanda, Angola; Department of Physiology, Faculty of Medicine, University Agostinho Neto, Luanda, Angola; Department of Physiology, Faculty of Medicine, University Agostinho Neto, Luanda, Angola; Department of Physiology, Faculty of Medicine, University Agostinho Neto, Luanda, Angola; Department of Physiology, Faculty of Medicine, University Agostinho Neto, Luanda, Angola

**Keywords:** salt intake, behavioural, medical students

## Abstract

**Background:**

Levels of salt consumption and its awareness among medical students in Angola remain insufficiently studied. This study determined salt intake and assessed medical students’ knowledge, attitude and behaviour regarding salt consumption.

**Methods:**

Were collected 24-hour urine samples from a random sample of 123 undergraduate medical students aged 17–43 years who were studying at the University of Agostinho Neto in Luanda. Their knowledge, attitude and behaviour regarding dietary salt were surveyed. Socio-demographic, clinical and anthropometric data were collected.

**Results:**

Average salt intake was 14.2 ± 5.1 g/day, without significant difference between genders (*p* = 0.221). In total, 96.7% consumed over 5 g/day, but only 6.5% of participants were aware of their excessive salt intake. The majority knew about salt-related health consequences and 45.5% reported they controlled their salt intake.

**Conclusions:**

This study indicated a high salt intake and inadequate behaviour regarding dietary salt consumption among medical students studying at the University of Agostinho Neto. This highlights the need for nutritional education to improve their dietary habits and future role in counselling.

## Abstract

High salt (sodium chloride) consumption is an important determinant of high blood pressure and cardiovascular risk. According to World Health Organisation (WHO) statistics, over 80% of cardiovascular disease (CVD) deaths take place in low-and middle-income countries, and elevated blood pressure levels were a major cause of these CVD deaths in those countries.^1^ Lifestyle factors such as unhealthy diet, physical inactivity, tobacco use and harmful use of alcohol have been considered the most important behavioural risk factors for heart disease and stroke.^2^

Among dietary factors, high salt intake has been the most strongly associated with raised blood pressure and increased risk of stroke and CVD.^3^ Therefore dietary sodium restriction has been recommended as a non-pharmacological approach to blood pressure lowering,^4^-^6^ and for the prevention and control of non-communicable diseases at the population level.^7^,^8^

Cumulative evidence has shown that even a modest reduction in salt intake was associated with blood pressure lowering and therefore with a significant reduction in incidence of cardiovascular events.^9^-^12^ Furthermore, data from the most recent systematic review and meta-analyses has shown the benefit of lowering sodium intake in apparently healthy adults and children,13 and in both hypertensive and normotensive individuals, irrespective of gender and ethnic group.^9^

Since hypertension is associated with CVD worldwide, a public health intervention to reduce high blood pressure must target the role of lifestyle, particularly reduced sodium intake.^7^ Therefore, several countries have initiated strategies to reduce dietary salt intake in the general population by a combination of various procedures such as public education, food labelling, and collaboration with the food industry to reduce the salt content of processed food.^14^

Among sub-Saharan African countries, only Nigeria and South Africa have developed dietary guidelines regarding salt intake.^15^ Recently, the South African government implemented important specific legislation towards decreasing salt intake in the population by reducing sodium content of processed foods by industries.^16^ Therefore, the current public health recommendation is that countries should launch national initiatives to reduce the over-consumption of salt as part of non-communicable disease prevention and healthy nutrition policies for limiting salt intake to less than 5 g/day for the general population including children.^7^ Despite of this guideline, however, high sodium intake remains prevalent around the world, with average daily salt intake varying from 5 to 18 g/day per person.^17^

Although processed foods have been found to be the principal source of excessive dietary salt intake,^18^ sources of dietary sodium vary largely worldwide and may be influenced by cultural context and dietary habits of the population.^19^ In sub-Saharan African countries experiencing demographic and epidemiological transition, the rapid rise in prevalence of CVD (chiefly hypertension) has been attributed to lifestyle change, including high dietary sodium intake.^20^,^21^ However, consistent data from studies on risk factors are lacking for the majority of these countries.

With regard to Angola, available data from a cross-sectional study reported a high prevalence of multiple cardiovascular risk factors, such as hypertension, sedentary lifestyle, electrocardiographic left ventricular hypertrophy,^22^ and high rate of the metabolic syndrome^23^ in an apparently healthy middle-aged population of university public employees living in urban and peri-urban areas.

Determining the level of sodium intake in the population is crucial to establish intervention strategies and policy on reduction of sodium intake. For medical students in particular, it is very important to assess their awareness regarding dietary salt intake, since they are the future providers of healthcare information for the counselling of people about the need to reduce salt consumption. The aim of this study was to determine salt intake and to assess the knowledge, attitude and behaviour regarding dietary salt among medical students.

## Methods

Participants were undergraduate medical students randomly recruited from a population of 625 students listed in 2013 at the Faculty of Medicine of the University Agostinho Neto (FMUAN) in Luanda, Angola. Due to limited resources, the study was planned to collect 24-hour urine samples from 30% of the students (*n* = 188) representative of the student population. Data were collected from September to October 2013 in the Department of Physiology of FMUAN.

The protocol of the study was in agreement with the Declaration of Helsinki and approved by the institutional review board. Each participant gave informed, written consent, and no compensation was given for participation in the study.

For recruitment, the academic secretary of FMUAN provided a list of all students from first to fifth year. We randomly selected 30% from each year and contacted them by phone one day later to invite them to participate in a cross-sectional survey on ‘cardiovascular risk factors’. A participant information sheet and a consent form explaining the purpose and protocol of the study were also provided. If participation was declined, another student from the same academic year, gender and age group was contacted.

Thus 188 students were randomly selected from 625 registered students, and 153 agreed to participate. Of these, seven subjects were excluded as they met the exclusion criteria [being pregnant (*n* = 1), having a history of hypertension or taking anti-hypertensive medication (*n* = 6)]. A further 23 students were excluded from the analyses due to incomplete 24-hour urine collections [i.e. 24-hour urine volume ≤ 500 ml (*n* = 5); urine loss more than once in 24 hours (*n* = 7), timing of the urine collection less than 23 hours (*n* = 11)]. Final analyses were undertaken on 123 participants (54 men and 69 women) with valid urine collections.

## Data collection

A standardised questionnaire was administered for each participant to obtain demographic data and information on medical history, smoking habits, intake of alcohol, use of medication and physical activity. Participants’ awareness of a diagnosis of hypertension was based on having previously been informed on the diagnosis of the condition, receipt of therapy for it, or an awareness of the purpose of antihypertensive therapy. Relevant knowledge, attitude and behaviour on dietary salt were assessed using a standardised questionnaire from the WHO.^24^

Participants were classified as non-smokers (never and ex-smokers) and current smokers (daily and occasional smokers). Alcohol consumption was assessed on their answer to the question about consumption of alcoholic beverage (‘yes/no’).

Physical activity was assessed with the following two questions: ‘Do you currently participate in any regular physical activity for leisure (‘yes/no’)? If ‘yes’, what frequency per week? Participants were classified as sedentary or inactive if they answered ‘no’ to the first question or reported a frequency of regular physical activity less than three days per week. They were considered active if they reported regular physical activity at least three days a week of at least 30 minutes or more per session.

## Study protocol and laboratory tests

Clinical examinations were performed between 08:00 and 12:00 in temperature-controlled rooms (22–23°C) after a 10- to 12-hour fast. Participants were asked to refrain from smoking, physical exercise and caffeinated beverages before the visit.

Venous blood samples were obtained from the forearm using standard techniques and processed immediately with commercially available kits (BioSystems SA, Costa Brava 30, Barcelona, Spain) for determination of levels of serum triglycerides, total cholesterol, glucose, creatinine and uric acid. Biochemical parameters were analysed using enzymatic methods with a spectrophotometer (BioSystems BTS-350, Costa Brava 30, Barcelona, Spain). Diabetes was defined as a fasting glucose level ≥ 126 mg/dl (7 mmol/l) or the use of antidiabetic drugs.^25^

Urine was collected during the 24-hour period preceding the clinic visit. Participants were asked to collect all urine they passed during a 24-hour period starting from the second urination on the morning of the collection day, and ending with the first urine passed the following morning. In order to maximise a correct 24-hour urine collection, participants were asked to collect their samples from Sunday 7:00 to Monday 7:00.

Participants were also asked to note on the record sheet the start and finish times of their urine collection, if some urine was lost, and any medication taken during the collection period. Females were encouraged to collect their urine on non-menstruation days.

For the collection, participants were provided with urine collection kits and standardised written instructions on how to collect and handle urine. The urine collection kit comprised five-litre plastic bottles with screw caps to serve as the collection container for urine; two-litre plastic bottles with screw caps for collections made away from home; one-litre plastic jugs; one funnel and a plastic carrier bag for transporting the equipment from home.

Participants were instructed to pass urine into the one-litre plastic jug, and then pour the sample into the five-litre container using the funnel provided. Plastic bags were provided to carry the equipment (including a smaller two-litre collection container) if participants were not at home for some of the collection period.

The validity of 24-hour urine collection was verified by a combination of three criteria. Urine samples were considered incomplete for a 24-hour collection period and excluded from analysis if: (1) there was more than one self-reported loss of a urine sample; (2) a 24-hour urine sample measured at the laboratory was ≤ 500 ml/day; (3) timing of the urine collection was less than 23 hours or more than 25 hours.

After validation, a sample of 60 ml was centrifuged at 3 500 rotations/minute, using a Sigma 2-6E device (Germany), before aliquots were sampled. The urine was transferred in duplicate into screw-top plastic test tubes. The aliquots were kept in a freezer within 24 hours of collection and stored at –15ºC until analysed in the laboratory at the Department of Biochemistry of FMUAN.

Urinary sodium and potassium concentrations in the aliquots were measured using the ion-selective electrode method on a Medica Easylyte Plus Na/K/Cl analyser (Netherlands). Sodium (Na) was converted from millimoles (mmol) to grams by dividing by 17 and the conversion from sodium to salt (sodium chloride) was made by multiplying by 2.542, as previously proposed.^26^

## Anthropometric and blood pressure measurement

Weight and height were measured using a digital electronic balance equipped with a digital stadiometer (SECA, GmbH & Co, Germany; range 0.1–250 kg, precision 50 g and range 110–200 cm, precision 0.1 cm, respectively). Body mass index (BMI) was calculated as the weight divided by the square of the height (kg/m^2^). According to BMI values, individuals were classified as overweight (25.0–29.9 kg/m^2^) and obese (≥ 30.0 kg/m^2^).^27^

The waist and hip circumferences were measured with participants in a standing position using a non-extending 1-cm-wide measuring tape. The waist circumference was measured at the end of normal expiration, at the midpoint between the lower border of the rib cage and the top of the iliac crest, and recorded to the nearest 0.1 cm.

Blood pressure and heart rate were measured in triplicate after five minutes of resting in a seated position, using a validated, automated digital oscillometric sphygmomanometer (Omron 705CP, Tokyo, Japan). The readings were repeated at three-minute intervals. The mean of the last two readings was recorded. Hypertension was defined as systolic blood pressure ≥ 140 mmHg, and/or diastolic blood pressure ≥ 90 mmHg, and/or the use of antihypertensive drugs.

A standard 12-lead electrocardiogram (ECG) recorded at rest for each participant, using a computerised device (Schiller AT-10 EKG, Baar, Switzerland). Each ECG was assessed by an experienced observer who was blinded to other clinical characteristics of the participants.

## Statistical analysis

The normality of the data was checked using the Kolmogorov–Smirnov test. Continuous variables are reported as mean ± standard deviation or median and interquartile range (25th – 75th percentile). These variables were compared by gender using the independent samples *t*-test or Mann–Whitney test for normally or non-normally distributed data, respectively. Categorical variables were expressed as proportions and compared using the chi-square test or Fisher’s exact test if appropriate. Data were analysed using SPSS software, version 13.0 (SPSS Inc, Chicago, IL). A two-tailed *p* < 0.05 was considered statistically significant.

## Results

The response rate for the random sample was approximately 68% (123/181) of the planned study sample after excluding subjects with potentially confounding factors that could influence urinary excretion of sodium and potassium. Of the 123 participants, the mean age was 22.6 ± 4.3 years (range 17–43), and more were women (56.1%) with a similar age to the men. The characteristics of the population are presented in Table 1.

**Table 1:** Characteristics of the participants by gender

*Characteristics*	*All (n = 123)*	*Men (n = 54)*	*Women (n = 69)*	*p-value*
Number (%)	123 (100)	54 (43.9)	69 (56.1)	0.245
Age (years)	22.6 ± 4.3	22.9 ± 4.4	22.5 ± 4.3	0.595
Weight (kg)	60.6 ± 13.1	64.5 ± 13.6	57.6 ± 11.8	0.003
Height (cm)	165.6 ± 7.8	170.3 ± 7.4	162.0 ± 6.1	< 0.001
WC (cm)	72.4 ± 9.9	74.1 ± 10.6	71.0 ± 9.2	0.088
HC (cm)	91.7 ± 10.4	90.2 ± 10.4	92.9 ± 10.4	0.150
BMI (kg/m^2^)	22.0 ± 3.9	22.1 ± 3.5	21.9 ± 4.2	0.819
SBP (mmHg)	113.8 ± 11.4	119.9 ± 11.6	109.6 ± 9.4	< 0.001
DBP (mmHg)	68.1 ± 7.5	67.6 ± 7.4	68.5 ± 7.6	0.501
Heart rate (bpm)	75.0 ± 11.0	72.0 ± 11.0	78.0 ± 9.0	0.002
Glucose (mg/dl)	90.3 ± 11.1	89.1 ± 12.4	91.2 ± 9.9	0.306
(mmol/l)	(5.01 ± 0.62)	(4.95 ± 0.69)	(5.06 ± 0.55)	
Creatinine (mg/dl)	0.96 ± 0.13	1.07 ± 0.10	0.88 ± 0.09	< 0.001
(μmol/l)	(84.86 ± 11.49)	(94.59 ± 8.84)	(77.79 ± 7.96)	
Uric acid (mg/dl)	4.8 ± 1.2	5.5 ± 1.0	4.2 ± 1.0	< 0.001
TC (mg/dl)	171.7 ± 36.4	175.5 ± 39.7	168.8 ± 33.6	0.311
(mmol/l)	(4.45 ± 0.94)	(4.55 ± 1.03)	(4.37 ± 0.87)	
TG (mg/dl)	79.3 ± 36.7	79.2 ± 36.3	79.5 ± 37.4	0.962
(mmol/l)	(0.9 ± 0.41)	(0.89 ± 0.41)	(0.9 ± 0.42)	
Hypertension, *n* (%)	4 (3.3)	3 (5.6)	1 (1.4)	0.203
Diabetes, *n* (%)	1 (0.8)	1 (1.9)	0 (0.0)	0.256
Overweight, *n* (%)	17 (13.8)	8 (14.8)	9 (13.0)	0.551
Obesity, *n* (%)	4 (3.3)	1 (1.9)	3 (4.3)	0.001
Sedentary, *n* (%)	97 (78.9)	35 (64.8)	62 (89.9)	0.001
Alcohol intake, *n* (%)	19 (15.4)	12 (22.2)	7 (10.1)	0.132

Values are means ± standard deviation or percentages. WC, waist circumference; HC, hip circumference; BMI, body mass index; SBP, systolic blood pressure; DBP, diastolic blood pressure; TC, total cholesterol; TG, triglycerides.

When compared with women, men had significantly higher mean values for weight, height and systolic blood pressure, and higher levels of blood creatinine and uric acid. Women had significantly higher heart rate values compared to men. The proportion of subjects with obesity and a sedentary lifestyle was significantly higher in women than men. There was no significant difference between men and women regarding the prevalence of hypertension, diabetes, obesity and alcohol consumption. None of the participants reported current or past smoking.

Participant’s answers to the questionnaire regarding their knowledge, attitude and behaviour on dietary salt are shown in Table 2. The majority of participants stated that salt was always added in preparing food at home, and rarely or sometimes added to food at the table. It was also observed that almost all participants knew that a high-salt diet could cause health problems, and 91.1% of them recognised the importance of reduced salt in the diet. However, less than half of the participants (45.5%) were aware of their high dietary sodium intake, and most reported a preventative measure was the avoidance of adding salt at the table.

**Table 2:** Knowledge, attitude and behaviour on dietary salt

*Question*	*Total n (%)*
Is salt added in cooking the food that you eat at the home?	
Never	0 (0.0)
Rarely	1 (0.8)
Sometimes	0 (0.0)
Often	19 (15.4)
Always	103 (83.7)
How much salt do you think you consume?	
Far too much	4 (3.3)
Too much	4 (3.3)
Just the right amount	69 (56.1)
Too little	31 (25.2)
Far too little	2 (1.6)
Don’t know	13 (10.6)
Do you add salt to food at the table?	
Never	30 (24.4)
Rarely	46 (37.4)
Sometimes	40 (32.5)
Often	2 (1.6)
Always	5 (4.1)
Do you think that a high-salt diet could cause a health problem?	
Yes	122 (99.2)
No	0 (0.0)
Don’t know	1 (0.8)
How important to you is lowering the salt/sodium in your diet?	
Not at all important	2 (1.6)
Somewhat important	9 (7.3)
Very important	112 (91.1)
Do you do anything to control your salt or sodium intake?	
Yes	56 (45.5)
No	63 (51.2)
Don’t know	4 (3.3)
If answered ‘yes’, what do you do to control your salt intake?	
Avoid/minimise consumption of processed foods	4 (7.1)
Look at the salt or sodium labels on food	0 (0.0)
Do not add salt at the table	47 (83.9)
Buy low-salt alternatives	2 (3.6)
Buy low-sodium alternatives	1 (1.8)
Do not add salt when cooking	1 (1.8)
Use spices other than salt when cooking	0 (0.0)
Avoid eating out	1 (1.8)

Less-reported measures were: avoidance or minimising salt intake, use of low-sodium or low-salt alternatives, avoidance of adding salt when cooking, and avoiding eating out. Unexpectedly, none of the participants reported the habit of reading food labels to see the sodium content before consumption. Similarly, our participants were unaware of the possibility of using spices with lower sodium content as a salt substitute in cooking.

When participants were asked their perception of the amount of salt they were consuming, the majority of them classified their own level of salt consumption as ‘just right’ or ‘too little’. Only 6.5% of participants recognised they consumed salt excessively.

Table 3 shows means (SD) and medians (25th and 75th percentiles) of urine volume, urinary sodium, urinary potassium and salt intake, according to gender. Compared to women, men had significantly higher mean values for urine volume and sodium-to-potassium ratio. There was no statistically significant difference between men and women for urinary sodium and potassium concentration and daily salt intake. The proportion of participants exceeding the limit of 5 g/day of salt in the overall population was 96.7%, without a gender difference.

**Table 3:** Mean and median values of urinary data according to gender

*Variables*	*All (n = 123)*	*Men (n = 54)*	*Women (n = 69)*	*p-value*
Urine volume (ml/d)				
Mean ± SD	1429.7 ± 649.0	1579.0 ± 738.5	1312.8 ± 546.7	0.023
Median (25th, 75th pc)	1320 (900, 1800)	1443.5 (915, 2152.5)	1250 (870, 1645)	
U Na (mmol/l)				
Mean ± SD	94.8 ± 34.4	99.2 ± 37.4	91.3 ± 31.7	0.221
Median (25th, 75th pc)	95.2 (65.1, 116.0)	102.8 (63.7, 122.3)	92.6 (66.7, 113.8)	
U K (mmol/l)				
Mean ± SD	33.9 ± 13.6	33.0 ± 15.9	34.7 ± 11.6	0.496
Median (25th, 75th pc)	33.3 (24.9, 42.9)	29.0 (22.0, 39.4)	34.7 (25.4, 43.5)	
Na:K ratio				
Mean ± SD	3.0 ± 1.2	3.3 ± 1.3	2.8 ± 1.0	0.029
Median (25th, 75th pc)	2.7 (2.2, 3.5)	2.8 (2.3, 4.0)	2.7 (2.1, 3.3)	
Salt intake (g/d)				
Mean ± SD	14.2 ± 5.1	14.8 ± 5.6	13.7 ± 4.7	0.221
Median (25th, 75th pc)	14.2 (9.7, 17.3)	15.4 (9.5, 18.3)	13.8 (10.0, 17.0)	
High salt intake				
(> 5 g/d), *n* (%)	119 (96.7)	53 (98.1)	66 (95.7)	0.439

SD, standard deviation; U Na, urinary sodium; U K, urinary potassium; Na:K, sodium-to-potassium ratio; 25th, 75th pc, 25th and 75th percentiles.

## Discussion

The purpose of this study was to determine salt intake so as to assess the knowledge, attitude and behaviour regarding dietary salt in a representative sample of medical students. The main findings were a high average daily salt intake and inadequate behaviour regarding dietary salt consumption in the majority of participants.

The level of salt intake seen in this study was more than two-fold the maximum internationally recommended limit,^7^ indicating a salt-rich diet consumed by our participants. This finding corroborates with that reported for the general population worldwide.^7^,^17^,^19^ Despite the known relationship between salt intake and blood pressure levels, data on salt consumption based on properly collected 24-hour urine samples are lacking for young medical students.

Participants for this study were randomly selected from a population of medical students and we used the ‘gold-standard’ 24-hour urine method to assess salt intake. Their knowledge, behaviour and attitudes on dietary salt intake were also assessed using a standardised WHO questionnaire.^24^

Salt intake was estimated using sodium concentration in 24-hour urine samples, and checks for completeness of 24-hour urine collection were based on a combination of self-reported urine loss, 24-hour urine volume measured at the laboratory, and the recorded timing of urine collection. These criteria enabled us to exclude 15% of the urine samples assumed to be incomplete collections for the 24-hour period, with the majority of them excluded due to incomplete timing of the urine collection. The validated urine samples were therefore 80.4% of the total collected.

Identifying the main source of dietary salt is important to control high salt intake in the population. Therefore, behavioural change in the use of salt is one of the strategies recommended to reduce high salt intake in contexts where most sodium intake comes from salt added during cooking or at the table at home.^7^

From the survey, we found that almost all our participants were aware of the health consequences of a high-salt diet, and reported more frequently eating food with salt added during cooking in their homes, and less frequently eating food with salt added at the table. However, less than half the total participants reported being aware of their high sodium intake, and the majority (83.9%) reported mainly avoiding adding salt to food at the table.

As previously reported,^28^ there is a tendency for individuals to perceive their dietary quality as good, even in the presence of results of an objective measure showing opposite results. Therefore, although it is difficult to know the exact amount of salt added to food at the table or in cooking, we found that contrary to the high urinary sodium values found, the majority of our participants classified their own level of salt consumption as ‘just right’ or ‘too little’, indicating a misperception of the amount of salt they were eating. This gap between the self-perceived and actual quality of a diet has been attributed to the inability of individuals to perceive their own dietary salt imbalance,^29^ therefore leading to an unrecognised high salt intake.

On the other hand, it has also been observed that some people have a taste preference for high-sodium foods,^7^ which leads to an inadequate perception of the amount of salt they are consuming. Of concern is that although our participants were medical students and future educators in public health, none of them reported the habit of reading the labels of processed foods to see the sodium content before consumption. Although sources of dietary sodium vary largely worldwide,^7^ a high amount of sodium has been found in processed foods,^30^,^31^ which are the main sources of dietary salt.

A high-potassium diet has many benefits for health. As previously reported,^32^ an increase of 42 mmol of potassium per day is associated with a 21% reduced risk of stroke. In our study, the average potassium intake was lower than the recommended value of approximately 90 mmol per day.^33^ Considering that the potassium excreted in 24-hour urine comes from the diet, the findings of lower values of urinary potassium in our participants suggest an unhealthy diet, in particular a poor consumption of fresh vegetables and fruits.

It has been advised that a healthy diet should provide enough content of potassium to achieve the molar ratio of sodium to potassium of approximately one to one.^33^ We found a ratio of three to one, confirming a high dietary salt intake in the majority of our participants. Although the proportion of subjects classified as having hypertension was low, there is a potential risk for early blood pressure in this young population if the current level of salt intake is maintained.

With regard to other classic cardiovascular risk factors, we found a high prevalence of physical inactivity and 15% of participants reported alcohol intake, but a low prevalence of hypertension, diabetes and obesity. The high prevalence of physical inactivity seen in this study is similar to the findings of a study that enrolled university students from developed and developing countries, in which physical inactivity tended to be higher among students from developing countries.^34^

A positive finding in our study was that none of our participants reported smoking. This result may reflect a possible cultural difference regarding smoking among young people from different countries.

The unsatisfactory behaviour regarding dietary salt seen in this study may reflect the fact that because our students were aware of their current health status, they did not worry about their salt intake and therefore did not perceive their high risk for the development of health-related consequences.

The main limitation of the study was that our sample was not representative of a national student population. Despite the small sample size, the strength of this study was that a possible selection bias was minimised by randomly selecting the students from the overall student body.

The complete 24-hour urine collection provided an estimation of salt and potassium consumption, reflecting the daily pattern of nutrient intake by our participants. Beyond the measurement of the amount of salt consumption, the study also included a survey on awareness and attitude regarding dietary salt, including discretionary salt use (i.e. cooking or at the table), which are important elements in finding the main source of salt consumed by our participants.

Overall, our findings suggest urgent educational action is needed to target behavioural change on dietary salt habits and other health-risk behaviour of the students. This is required for early prevention of the development of chronic non-communicable diseases.

## Conclusion

The study indicates a high salt intake among medical students, with a misperception of their level of salt intake, and insufficient attitude and behaviour regarding control of salt intake. These results justify urgent nutritional education to upgrade their knowledge for appropriate behaviour aiming at reducing their salt intake and preparing them for their future role in community counselling.
